# Prosocial Orientation of Russians During the COVID-19 Pandemic: Caring for Others and Yourself

**DOI:** 10.3389/fpsyg.2021.629467

**Published:** 2021-07-08

**Authors:** Pavel A. Kislyakov, Elena A. Shmeleva

**Affiliations:** Department of Psychology, Russian State Social University, Moscow, Russia

**Keywords:** prosocial orientation, prosocial behavior, self-care, care for others, COVID-19 pandemic, psychological safety

## Abstract

To mitigate the potentially devastating effects of the COVID-19 pandemic, it is vital to identify psychosocial and moral resources. The care, preservation, protection, and well-being of social communities are attributes of prosocial behavior that can be such a resource. The purpose of the study is to identify the features of prosocial orientation of Russian youth during the COVID-19 pandemic, as well as to identify strategies for prosocial behavior during the COVID-19 pandemic. The sample consisted of 447 people. The study was conducted in May 2020 in the form of an online survey of subjects using Google Forms (“Moral Foundations Questionnaire method” and “Portrait Values Questionnaire”). The research made it possible to establish that Russians were dominated by norms of care, fairness, purity; values of benevolence-universalism, security, and self-direction. During the COVID-19 pandemic, the prosocial orientation of Russians may manifest itself in the following behavioral strategies: proactive prosocial strategy of “caring for others” (true altruism, expressed in forms of volunteering, helping a stranger, and charity despite the risk of contracting a coronavirus infection); egoistic strategy of prosocial behavior “self-care through caring for others” (volunteering based on self-development; helping a stranger to improve your own psychological well-being); conventional prosocial strategy “self-care” (self-isolation and preventive behavior). In the long run, it is necessary to identify personal and environmental resources that allowed people to effectively implement a prosocial self-isolation strategy during the COVID-19 pandemic, as well as various forms of volunteerism.

## Introduction

Changing of our collective behavior is crucial to saving lives in the face of a new infectious disease. To mitigate the potentially devastating effects of the COVID-19 pandemic, it is vital to identify psychosocial and moral resources (Wolf et al., 2020). The care, preservation, protection, and well-being of social communities (another person, team, social organization, etc.) are attributes of prosocial behavior that can be such a resource. Addressing the altruistic and prosocial orientation of an individual can be an important aspect of response to social dilemmas during the pandemic (Van Bavel et al., 2020).

Psychologists, sociologists, anthropologists, biologists have noted that prosocial behavior is a central aspect of human life and the focus of research in the natural and social sciences (Zaki and Mitchell, 2013). Prosocial behavior refers to a broad category of acts that are generally beneficial to other people or society and includes such behaviors as cooperating, resource sharing, and helping (Penner et al., 2005; Twenge et al., 2007). Prosocial orientation of an individual is characterized by a system of motivations associated with activities for the benefit of others and society as a whole, with a sense of duty, responsibility to the group or society. In case of prosocial orientation, the individual is identified with the group. The constant threat of natural disasters and related shocks has probably shaped our prosocial motives throughout human evolution (Vardy and Atkinson, 2019).

Genetic characteristics determine prosocial behavior at the biological level and its focus is to preserve the human race. Empathy is the emotional basis for prosocial behavior. At the social level, prosocial behavior is supported by such norms (motives) as a social responsibility norm (encourages a person to help those who need it), a social reciprocity norm (people should help those who help them), a social fairness norm (rules on fair and just distribution of resources) (Aronson et al., 2010). Based on an interdisciplinary approach and anthropological research results, the intentionality of morality in the relationship between self-caring and caring for others has been established, revealing the paradox of self-caring: “the self-care ability develops when we care for others” (Bénabou and Tirole, 2005; Harbaugh et al., 2007; Barile et al., 2015; Kozlova and Kosheleva, 2015).

Human values and moral norms are essential in creating the possibility that people obey behavioral demands and display prosocial behavior. There is evidence that values of self-transcendence and security (Bardi and Schwartz, 2003; Fischer and Smith, 2006; Sagiv et al., 2017; Schwartz et al., 2017; Wolf et al., 2020), as well as moral identity (including moral norms, thinking, and emotions) (Hardy et al., 2015; Ding et al., 2018; Patrick et al., 2018; Gotowiec and Mastrigt, 2019; Lebedeva, 2019) predict the prosocial behaviors (charity, voluntary assistance, cooperation, empathy).

According to the theory of moral grounds, the prosocial orientation can be defined using the following moral grounds: “care” (care for the surrounding people and environment, developed ability for empathy and interpersonal interaction), “fairness” (“honesty”) (values of equality of all people, honesty and fairness in relations with others), “loyalty” (“collectivism”) (loyalty of a person to a social group, with which he identifies himself), “respect for authority” (“power”) (tendency to worship and subordinate to authority, observance of traditions, rules of conduct, public order), “purity” (“holiness”) (value of religious beliefs, loyalty to ideals of moral, and physical purity) (Graham et al., 2013).

Thus, the values and moral norms shared by society may be a key binding factor in promoting the collective prosocial orientation necessary during the COVID-19 pandemic. It is the value-semantic component of the prosocial orientation that determines the main relations of a person to the world and to himself. In this intention, the conditionality of prosocial behavior manifests itself. This relationship is the basis for the study of moral norms and value orientations, as well as preferred strategies of prosocial behavior during the transformation of social interactions caused by COVID-19 (Asmolov et al., 2020).

## Literature Review

In response to COVID-19, self-help groups and medical volunteers have become widespread in many countries (Booth, 2020; Holt, 2020; Unitus Europe European Philanthropy Social Investing Impact Hub, 2020; Yuan, 2020). In order to support people in terms of the pandemic, Russia has launched the “We Are Together” mutual assistance campaign, which includes medical, psychological, legal assistance, collection of donations to support the elderly, provision of healthcare facilities, and volunteer headquarters (My Vmeste, 2020).

The online magazine “BRICS Business Magazine” in April 2020 launched a media project “COVID-19-the Correct Answer” (https://covid.bricsmagazine.com/). The goal of the media project is to draw attention to professionalism, altruism, caring, and empathy, which in all its forms helped individuals, cities, countries, and the whole of humanity to win the fight against the pandemic. The site contains stories about the helping behavior, heroism and self-sacrifice of doctors, patients, scientists, teachers, politicians, civil servants, businessmen, law enforcement, military, civil activists, volunteers, journalists, and ordinary people (Press-Release.ru, 2020).

Sociological studies conducted in Russia reveal some contradictory data. On the one hand, Russians demonstrate their attraction to prosocial orientation and to a selfish one, on the other hand. Thus, according to the results of the Levada Center sociological survey, half of those polled, replying the question “What will happen to relationships between people in our country in the epidemic situation?,” chose the following answer: “People will take more care only about themselves and ‘their own'”; one-third replied “Nothing will change between people”; and only 17% supported the version “People will become more supportive of each other” (Levada-Centre, 2020).

In March–April 2020, the Institute of Psychology of the Russian Academy of Sciences conducted research on the attitude of Russians to the COVID-19 pandemic. The results of the research show that one-third to half of the respondents demonstrated a prosocial orientation («I forward information to my friends and acquaintance that can help them during the epidemic»–50%; «I'm willing to donate money to help the elderly who fell ill during the epidemic»–31%, etc.) or prosocial perception («If my family gets sick, I am sure they will get the required help from other people»–30%, etc.) of others for a number of indicators (Institute of Psychology RAS, 2020).

In March–April 2020, The Russian Center for Public Opinion Research conducted a study on Russians' awareness of volunteer activities during the spread of coronavirus infection and their readiness to provide volunteer assistance themselves. More than half of the Russians (61%) declared their readiness to provide volunteer assistance to people under quarantine, including single people. Every sixth Russian (15%) has already had to provide gratuitous help to elderly people or those who are under home quarantine due to the coronavirus («Inform surrounding people about the ways of coronavirus transmission and methods of its prevention»–19%; «Убирать подъезды, жилые помещения общего пользования» –13%; «Buy or produce your own protection and hygiene products»−11%; «Provide assistance in solving everyday problems»–8%; «Provide psychological help, support, psychological counseling»–7%, etc.).

In the face of a pandemic, a proactive strategy of prosocial behavior shall be a strategy of “caring for another person,” which can exist in the form of volunteering, charity, and situational help to a stranger. The proactive strategy bases on the ethics of “love for a distant” and “duty motives,” and is related to the ability to accumulate and use economically any kind of resources necessary to achieve time-distant life goals (Slabinskii and Voishcheva, 2016).

Sociological research data has generally reflected a tendency for volunteerism and charity in Russia in recent years. Thus, according to WCIOM sociological survey conducted in September 2019, 19% of Russians are regularly engaged in charity or volunteering, and 68% are ready to engage in charity or volunteering in future (WCIOM, 2019). We may assume in this regard that the motives for providing assistance during the COVID-19 pandemic are also primarily selfish and related to self-actualization, social skills development, and increase of social contacts, etc., despite the risk of contracting coronavirus infection (Kislyakov et al., 2019). Thus, the second strategy of prosocial behavior during the COVID-19 pandemic is the selfish strategy of “self-care through caring for others.” At the same time, mechanism of prosocial behavior realization can be mechanism of psychological protection (coping) and development.

Raposa et al. showed that participation in prosocial behavior could be an effective strategy for reducing the impact of stress on psycho-emotional status (Raposa et al., 2016). Dawans et al. proved in laboratory conditions that people who experienced acute social stress showed more prosocial behavior (trust, reliability, and sharing) (Dawans et al., 2012). Thus, the authors arrived at the conclusion that participation in prosocial behavior in response to stress can be a protective pattern. Krysko notes that prosocial behavior leads to good social well-being of a human through reflexing (Krysko, 2016). Luria et al. showed that the avoidance of uncertainty was a predictor of volunteering and donations at the individual level (Luria et al., 2019).

Finally, the third strategy for prosocial behavior in a pandemic is the “self-care strategy,” which manifests itself in the form of preventive or health-saving behavior (Wilson, 2018). The inextricable link between caring for others and self-care is evidenced by research on health-saving behaviors that consider self-care as an activity of individuals, families, and communities undertaken to promote health, prevent disease and restore health. Research of processes in which self-care acts as a strategy for preserving an individual's identity indicates a deep, value, and moral motivation for health-saving behavior (Kozlova and Kosheleva, 2015). For example, a study of the health-saving behavior of older people shows that individuals interpret self-care practices as a moral obligation to society (Roberto et al., 2005). Caring for one's own health, a person retains his independence as well as the ability to take care of others (Clarke and Bennett, 2013).

Kappes et al. found that in hypothetical scenarios for deciding whether to go to work when sick, the American and British participants in the experiment reported that they would be less willing to stay home when it was doubtful that they would infect a colleague. However, when going to work, at the risk of infecting an older colleague who has a serious illness, the participants reported that they would be more willing to stay home. Thus, focusing on the worst-case scenarios, even if they are unsure, may encourage people to make sacrifices for others (Kappes et al., 2018).

Preventive behavior during the COVID-19 pandemic should be linked to self-isolation and should aim to exclude or reduce their contact with others who may pose a threat to themselves or, conversely, to others, “to practice caring for their own being and that of others, to perform the work of the individual for their well-being—physical, mental, and spiritual—and to eliminate their own and others' disadvantage” (Magomed-Eminov, 2020; Wolf et al., 2020). Pfattheicher et al., conducting research in the UK, USA, and Germany, found that empathy (as the emotional basis for prosocial behavior) predicted and even increased motivation to observe the rules of physical distancing and wearing medical masks (Pfattheicher et al., 2020).

Most of the negative effects on the human psyche relate to forced restriction of liberty. The “self-care strategy” adoption through voluntary self-isolation helps to reduce stress. Therefore, authorities and social institutions should emphasize altruism in their choice of self-isolation (Kudryavceva, 2020). Awareness of prosocial behavior associated with observing the rules of social distance and hygiene forms a sense of collectivity and helps preventing mental disorders caused by self-isolation (Guo et al., 2020).

A research by psychologists at the Institute of Psychology of the Russian Academy of Sciences showed that 70% of respondents believed that salvation from the COVID-19 pandemic was the moral consciousness and responsibility of each person. About 70% of respondents are also aware of the importance of preventive behavior during the COVID-19 pandemic (self-isolation and wearing masks) (Institute of Psychology RAS, 2020). At the same time, as noted by Nestik, one of the reasons for observing the precautionary rules during the COVID-19 pandemic, along with fear of infection, is compassion for others (empathy) and solidarity with others (Nestik, 2020).

Dryhurst and colleagues completed a study aimed at measuring the COVID-19 risk perception index in 10 countries in Europe, America, and Asia. The index reflected people's perceptions of danger of the COVID-19 pandemic, the perceived likelihood that they themselves, their family members and friends would become infected with the virus, and the level of concern about the virus. The study found that prosocialism, expressed in recognition of the importance of doing something for the benefit of others and society, even to the detriment of personal interests, was more or less a predictor of awareness of the COVID-19 pandemic risks for people in all countries (from different cultures) (Dryhurst et al., 2020).

According to the Russian Public Opinion Research Center, four out of five Russians (81%) who participated in a sociological poll are self-isolating. Moreover, 76% have limited their contacts, stay at home or have gone to a faraway location (WCIOM, 2020).

As Leontiev notes, sociological researches allow to fix the change of attitude to many realities being on the surface of consciousness (Leontiev, 2020). Psychological research, on the other hand, deals with more stable mechanisms. In this regard, psychological research is required to study changes in prosocial orientation and to implement behavioral strategies based thereon during the COVID-19 pandemic. Our research is devoted to this problem solution.

Existing theoretical and empirical studies show, albeit indirectly, that first, people are shifting toward prosocialism during the COVID-19 pandemic, and secondly, different strategies of prosocial behavior based on caring for others and/or self-concern are possible (Pfattheicher et al., 2020; Wolf et al., 2020). In addition, we have formulated the following hypotheses based on the evidence that values define forms of prosocial behavior (Sagiv et al., 2017; Kislyakov et al., 2020; Wolf et al., 2020): (1) during the COVID-19 pandemic the Russians have shifts in values and morality toward prosocial orientation; (2) prosocial behavior during the COVID-19 pandemic could be characterized by three strategies: proactive “caring for others” strategy, selfish “self-care through caring for others” strategy, and conventional preventive “self-care” strategy.

## Materials and Methods

The sample consisted of 447 people (41% men, 59% women) aged 17 to 25 years (*M* = 20), university students in Moscow, Ivanovo, Kostroma, Yaroslavl; 57.7%—had experience in volunteer activities. A sampling is formed from young persons, because they are the most active and mobile part of society, involved in various social processes (including volunteering—as a form of prosocial behavior), and are faster to respond to the changes and adapt to them, including those related to the moral and value orientations.

In each city, one state university was selected, which implements various programs (sciences and humanities). All universities taught using distance learning, and students were studying at home. Anti-epidemic restrictions were in effect in the cities (cancellation of mass events, wearing of medical masks, etc.). The procedure of “convenience” sampling was used; the students voluntarily took part in the research for additional points in the academic ranking. The study was conducted in May 2020 in the form of an online survey of subjects using Google Forms. Students were sent an email to their personal e-learning accounts.

The prosocial orientation was assessed based on indicators of the moral norms development and value orientations.

The initial stimulus used for the survey was “*During the COVID-19 pandemic, it is important that people take care of the health and well-being of others. Care can be expressed in donations, participation in volunteer actions, psychological support, observance of social distance and rules of behavior, etc. Answer the questions given the COVID-19 pandemic situation*.”

The evaluation of the level of moral norms development was carried out with the help of Moral Foundations Questionnaire method (MFQ) (Heidt et al., adapted in Russian by Sychev et al., 2018). This methodology is based on the moral grounds classification developed by J. Heidt: “care,” “fairness,” “loyalty,” “respect for authority,” “purity.” A subject consented with 32 statements on a 6-point scale—measured from “1—not at all important/absolutely disagree” to “6—extremely important/absolutely agree,” embodying one or another moral value.

Value orientations were investigated using a shortened version of the “Portrait Values Questionnaire” methodology (PVQ-21) (Sh. Schwartz, adapted in Russian by Davidov et al., 2008). This methodology is based on the value classification developed by Schwartz and assesses seven typological value indices (scales): “security” (safety and stability of society, relations and oneself), “conformance-tradition” (containing actions and motives that may harm others and do not meet social norms), “self-direction,” “stimulation,” “hedonism,” “achievement-power” (social status, domination over people and resources), “care for people and nature” (benevolence, universalism). The subject evaluates 21 descriptions of people characterized by certain values on a 6-point scale—from “1—not at all like me” to “6—very similar to me.”

The resulting empirical data were processed using Friedman's two-factor dispersion analysis for related samples, hierarchical cluster analysis (intergroup bonding method), Mann-Whitney *U*-test, linear regression analysis (step method), Pearson's correlation analysis. Calculations were made based on the SPPS 23 statistical software package.

The study was conducted in accordance with the ethical code of The Russian psychological society, and the Protocol was approved by the Academic Council of the faculty of psychology of the Russian State Social University (Protocol No. 4 of 28.04.2020) and with the ethical standards of the World Medical Association Declaration of Helsinki. The questionnaire included the item “I confirm that I have read and understood the purposes, procedure, method, and possible inconveniences of participation in the research. I give my consent to participation in the research. I can give up or end the questionnaire at any time.”

## Results

The research revealed that for all scales of MFQ technique, the average values correspond to the average level of moral norms development (from 17 to 27 points). Quite high indicators of internal consistency of the questionnaire scales were confirmed (Cronbach's alpha > 0.7) (see Table 1). Friedman's two-factor rank dispersion analysis was used to identify the dominant moral norms in the sample under study.

**Table 1:** Comparative analysis of the moral norms dominance among Russians during the COVID-19 pandemic (MFQ).

**MFQ scales**	***M***	**Cronbach's alpha**	**Mean rank**	**Friedman test**
Care	26.06	0.755	3.86	χ^2^ = 500.52 *p* ≤ 0.001
Fairness	25.89	0.723	3.67	
Purity	23.85	0.719	3.01	
Loyalty	22.30	0.812	2.57	
Respect	20.46	0.764	1.89	

To identify the value orientations (indices) of Russians using the PVQ-21 method, an arithmetic mean (from 1 to 6) was calculated for each scale since the scales were measured in different ranges. The research made it possible to establish that for all scales of the PVQ-21 method, the average values correspond to the average level of formation of value indices (from 3 to 4 points). Quite high indicators of internal consistency of the questionnaire scales were confirmed (Cronbach's alpha > 0.7). Friedman's two-factor dispersion analysis was used to identify dominant value orientations in the sample under study (see Table 2).

**Table 2:** Comparative analysis of the value orientation dominance among Russians during the COVID-19 pandemic (PVQ-21).

**PVQ-21 scales**	***M***	**Cronbach's alpha**	**Mean rank**	**Friedman test**
Benevolence-universalism	4.39	0.801	4.95	χ^2^ = 388.39 *p* ≤ 0.001
Security	4.13	0.747	4.34	
Self-Direction	4.22	0.723	4.54	
Hedonism	4.12	0.784	4.31	
Stimulation	3.94	0.856	3.90	
Achievement-power	3.69	0.809	3.35	
Conformity-tradition	3.27	0.774	2.61	

**Figure 1:**
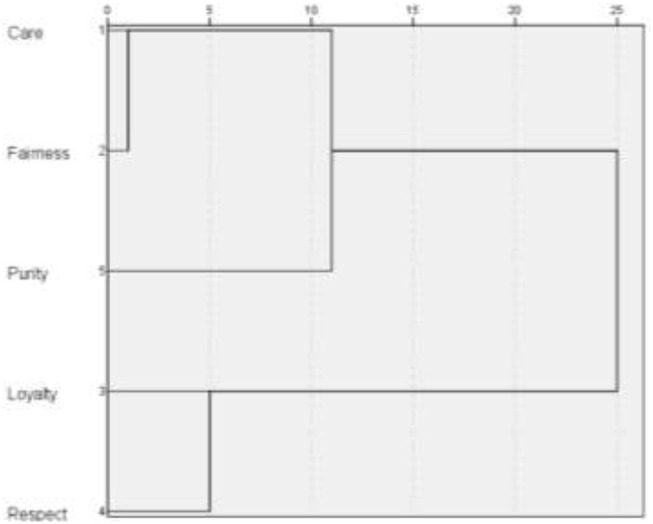
Classification of Russians' moral norms during the COVID-19 pandemic (MFQ). Dendrogram of hierarchical cluster analysis (intergroup communication method).

**Figure 2:**
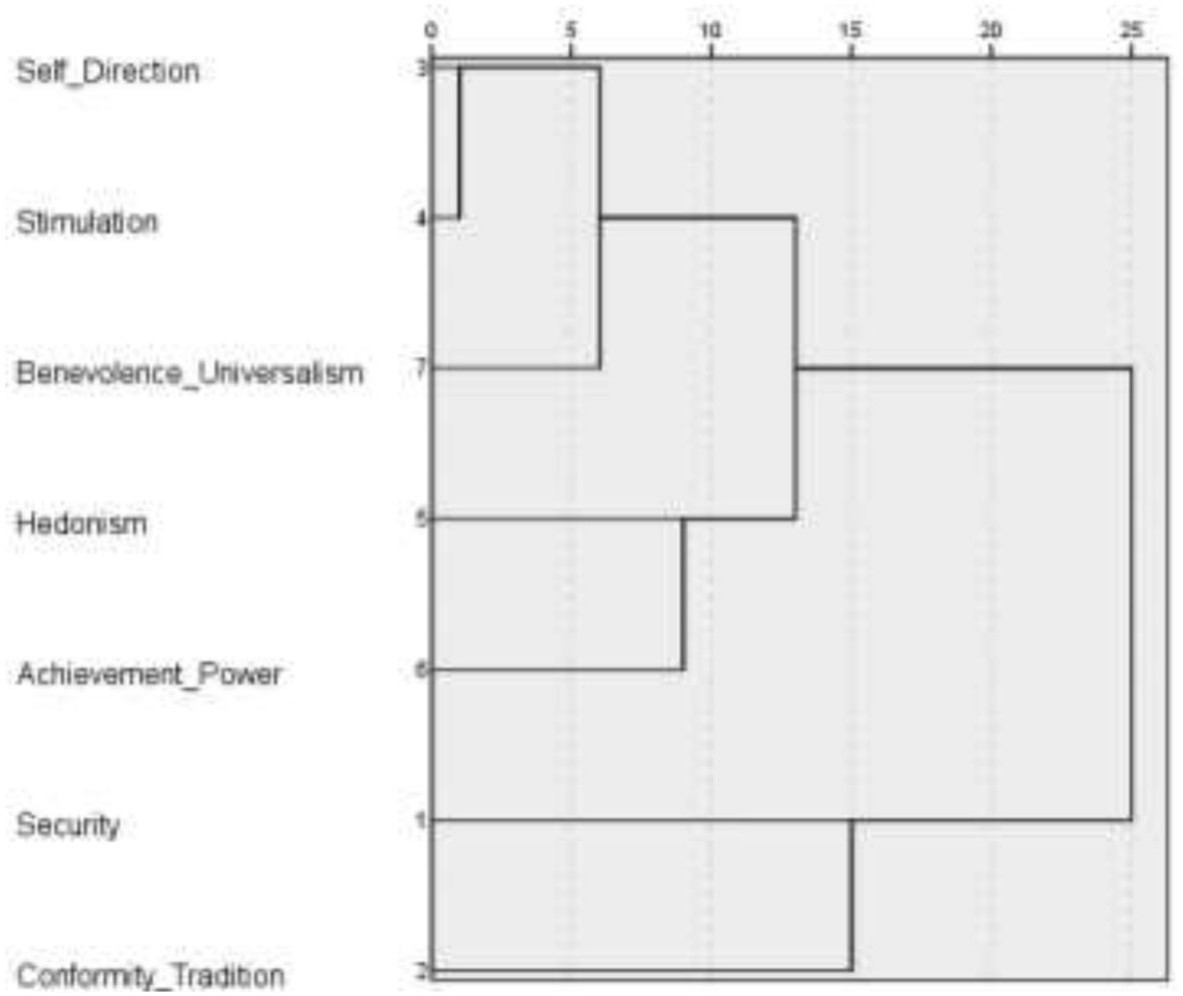
Classification of Russians' value orientation during the COVID-19 pandemic (PVQ-21). Dendrogram of hierarchical cluster analysis (intergroup communication method).

To test the second hypothesis of three prosocial behavioral strategies (“caring for others,” “self-care through caring for others,” and “self-care”) for Russians during the COVID-19 pandemic, a hierarchical cluster analysis (intergroup communication method) was conducted (see Figures 1, 2).

To study the impact of the “volunteer experience” on the prosocial orientation of Russians during the COVID-19 pandemic (according to indicators of moral norms and values orientation), we compared two groups using Mann-Whitney *U*-test (see Table 3).

**Table 3:** Comparison of Russians in terms of moral norms (MFQ) and value indices (PVQ-21), taking into account volunteer experience.

**Indicators**	**Mean rank**		**Mann-Whitney *U*-test**	***p***
	**Experienced in volunteering (*n* = 258)**	**Having no experience in volunteering** **(*n* = 189)**		
**MFQ scales**				
Care	239.37	203.02	20,416	0.003
Fairness	238.95	203.59	20,523.5	0.004
Loyalty	238.05	204.82	20,756	0.007
Purity	228.38	218.03	23,252	0.402
Respect	223.14	225.18	24,158	0.869
**PVQ-21 scales**				
Benevolence-universalism (Care)	236.63	206.76	21,123.5	0.015
Self-Direction	238.75	203.87	20,576	0.004
Stimulation	246.29	193.57	18,630	<0.001
Achievement-power	235.50	208.30	21,414.5	0.028
Hedonism	230.13	215.63	22,799.5	0.238
Conformity-tradition	214.82	236.54	22,011.5	0.078
Security	222.27	226.37	23,934	0.738

A linear regression analysis (step method) was performed to identify Russians' prosocial orientation predictors during the COVID-19 pandemic (see Table 4).

**Table 4:** Predictors of Russians' prosocial orientation during the COVID-19 pandemic.

**Predictors**	**β**	***p***	**Summary for the model**
**MFQ scales (“care” dependent variable)**			
Fairness	0.595	<0.001	*R^2^* = 0.676; *F* = 463.223. *p* < 0.001
Purity	0.310	<0.001	
**PVQ-21 scales (“benevolence-universalism” dependent Variable)**			
Self-direction	0.355	<0.001	*R*^2^ = 0.45; *F* = 90.304. *p* < 0.001
Conformity-tradition	0.275	<0.001	
Stimulation	0.179	<0.001	
Security	0.191	<0.001	

**Figure 3:**
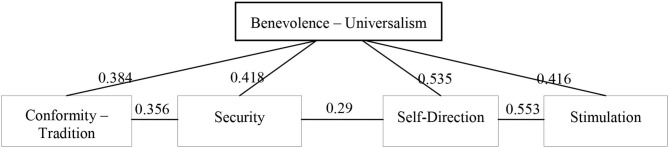
Value indices correlation Pleiades. Pearson correlation, Significance level *p* < 0.001.

Pearson's correlation analysis also revealed the existence of correlation relationships between the value indices of benevolence-universalism (care), conformism, security, stimulation, self-direction (PVQ-21) (see Figure 3).

## Discussion

The data in Tables 1, 2 show that Russians were dominated by norms of care, fairness, purity; values of benevolence-universalism, security, and self-direction.

The cluster analysis allowed to distinguish three classes (types) of social orientation of Russians during the COVID-19 pandemic (see Figures 1, 2).

The first type of social orientation can be characterized as “caring for others” proactive prosocial strategy. This type is defined by the moral norms of care, fairness and purity (MFQ) or the values of benevolence (universalism, fairness), self-discretion and stimulation (PVQ-21). Harvey and Erdos note that the psychological factors of risk-assisted behavior in an emergency area are altruism, heroism, and prosocial orientation of the individual (Harvey and Erdos, 2003). Studies conducted in Spain (Serrano-Montilla et al., 2021), Serbia (Dinić and BodroŽa, 2021), Canada, and the United States (Sin et al., 2021) showed that the health threat of COVID-19 predicted a tendency to express altruistic prosocial actions: anonymous helping behavior (Dinić and BodroŽa, 2021), volunteering and providing support (Sin et al., 2021), empathic care (Serrano-Montilla et al., 2021).

The second type of social orientation, defined by the norms of respect for power and loyalty (collectivism) (MFQ) or the values of security and conformism (PVQ-21), can be described as a conventional strategy. Conventional norms directly relate to the “self-care” prosocial strategy through respect for sanitary standards and norms of social distance. Campos-Mercade et al., 2021; Petrocchi et al., 2021 found that the motivation for self-isolating behavior can be altruistic. Ceylan and Hayran (2021) also note that compliance with restrictive measures is considered as prosocial behavior based on social responsibility. Kejselman calls introjection as a way to implement a prosocial behavior. The introjection mechanism helps to suppress conflict with the dominant worldview, and adapts a person to reality (Kejselman, 2016). One of the factors of prosocial behavior and caring for others in a crisis is the emerging sense of common identity and collectivism, which stem from a common experience of being in straitened circumstances (Drury et al., 2009; Cruwys et al., 2020; McKinley, 2020). Di Napoli et al. (2021) and Politi et al. (2021) found that prosocial attitudes during the COVID-2019 pandemic are driven by collective values and solidarity. Developing a sense of loyalty can help coordinate threat management efforts (Haslam and Reicher, 2006) and promote group commitment and social norms (Ellemers et al., 2002; Neighbors et al., 2010; Stevens et al., 2019). In doing so, the authorities (leaders) can have a significant impact on prosocial behavior by acting as role models (Schnall and Roper, 2012; Yang et al., 2018).

The third type of social orientation can be described as a selfish strategy “self-caring through caring for others,” defined by the values of hedonism and achievement-power (PVQ-21). Studies conducted in China have shown that prosocial coping with psychological pressures and stresses caused by the COVID-19 pandemic contributed to reducing mental health problems (Guo et al., 2020; Chong et al., 2021). Banerjee and Nair indicate that mutual help is an element of psychosocial intervention during the COVID-19 pandemic (Banerjee and Nair, 2020). However, there are studies showing that people with prosocial tendencies do not feel safe during the COVID-19 pandemic (Niemi et al., 2021).

Yamamoto psychologist of the Tokyo Mental Health, among the measures to minimize the psychological consequences of the pandemic, points out the importance of altruism, empathy, and Prosocial behavior not only about those who are important, but also about those who are in a difficult situation (it can be one short phone call and one email). Yamamoto notes that as social animals, we humans strive for contact, compassion, and concern for nature and others (Yamamoto, 2020).

Mahovskaya, replying to the question “How to behave in self-isolation due to the COVID-19 pandemic?” notes that those who help others survive in difficult periods live long generally (Mahovskaya, 2020). Magomed-Eminov in his recommendations on human behavior and activities in the COVID-19 pandemic contingency also notes the need to take care of loved ones, because taking care of others maintains a positive attitude in the person and enhances the meaning of life (Magomed-Eminov, 2020).

Leahy developed a scheme “What do I want to be during the COVID-19 pandemic?” which includes the position “I think about others and how I can help them” in the growth zone. Leahy offers the following recommendations during the COVID-19 pandemic: “Be supportive, ask for help and offer help! There is nothing more satisfying than to help a lonely person who is struggling or to help your loved ones, especially the elderly and the weak. Our feelings of belonging, gratitude, mutual support, and the substances that the body produces when we help others are the best immune assistants we have now” (Leahy, 2020). Thus, the strategy “I am for the World” becomes a resource for human development and psychological security (Fedosenko, 2020).

The conducted research has shown that people experienced in volunteering have more developed moral norms of care, fairness, loyalty as well as values of benevolence-universalism, self-direction, stimulation, and achievement-power (see Table 3).

Our correlation analysis and linear regression analysis (see Table 4, Figure 3) showed that the values of self-direction, stimulation, conformism, and security are the predictors of the value of benevolence-universalism (PVQ-21) (prosocial). Thus, the regression equation includes two behavioral strategies. The first proactive prosocial strategy relates to caring for others (autonomy and risk). Volunteers assisting those in need during the COVID-19 pandemic are ready to risk their health and well-being, and independently organize assistance and charity projects. The second conventional prosocial strategy is to take care of oneself and to observe sanitary and social norms of distance (conformism and security). Predictors of the moral norm of care (MFQ) are norms of fairness and purity. This prosocial strategy also relates to caring for others.

## Conclusion

One can expect prosocial behavior to manifest itself in different ways depending on individuals' group identity, psychological well-being, altruistic norms, and volunteer experience to the extent that different emergencies affect the human psyche.

Despite any cross-cultural differences, the global data convincingly show that it is useful to activate values of self-transcendence and security in order to motivate people's behavior to support the mitigation of the pandemic. To reduce any negative social and psychological consequences of the COVID-19 pandemic, the society and authorities should not rely on fear but on collective control, compassion, support, and solidarity. Studies show that cultures that are accustomed to prefer security to freedom are easier to coordinate in the face of a pandemic.

The results of numerous studies in social psychology, clinical psychology, personality psychology, and neuropsychology have shown that “Self” maturity becomes the basis for compassion and caring for the Other. The self-care ability develops when one cares for others. The Other becomes a necessary participant in the process of self-consciousness and the formation of one's own identity. In emergencies, prosocial behavior can be a coping strategy.

Values of self-direction, stimulation, conformism, and security were the prosocial predictors of Russian youth during the COVID-19 pandemic. Norms of fairness and purity define the moral norm of caring for others.

Based on the obtained data analysis, it has been established that during the COVID-19 pandemic, the prosocial orientation of Russians may manifest itself in the following behavioral strategies: proactive prosocial strategy of “caring for others” (true altruism, expressed in forms of volunteering, helping a stranger, and charity despite the risk of contracting a coronavirus infection); egoistic strategy of prosocial behavior “self-care through caring for others” (volunteering based on self-development; helping a stranger to improve your own psychological well-being); conventional prosocial strategy “self-care” (self-isolation and preventive behavior).

In the long run, it is necessary to identify personal and environmental resources that allowed people to effectively implement a prosocial self-isolation strategy during the COVID-19 pandemic, as well as various forms of volunteerism.

## Limitations

The resulting empirical results should be interpreted in light of several important limitations. First, it was a “convenience sampling.” Thus, temporal order and causality cannot be verified. Future studies should use some longitudinal structures for further examination of the regularities we have found. The sampling is not necessarily representative for the entire Russian society. Replication with the use of different sampling methods (gender, age, ethnicity, profession, etc.) is necessary. Future studies can confirm the reliability of the obtained results by analyzing various forms of prosocial behavior directly during the COVID-19 pandemic (volunteering, charity, membership in charity organizations, experience in helping a stranger, etc.) and personal characteristics (empathy, social identity, trust in the world, etc.).

## Data Availability Statement

Publicly available datasets were analyzed in this study. This data can be found here: https://www.europeansocialsurvey.org/about/country/russian_federation.

## Ethics Statement

The studies involving human participants were reviewed and approved by Academic Council of the faculty of psychology of the Russian State Social University (Protocol No. 4 of 28.04.2020). Written informed consent for participation was not required for this study in accordance with the national legislation and the institutional requirements.

## Author Contributions

PK conducted a comprehensive analysis of the state of research on the problem of prosocial behavior. ES justified the methodological basis of the study. Both authors conducted analytical work on the processing and analysis of the results of empirical research.

## Conflict of Interest

The authors declare that the research was conducted in the absence of any commercial or financial relationships that could be construed as a potential conflict of interest.
